# Lignin Degradation by *Klebsiella aerogenes* TL3 under Anaerobic Conditions

**DOI:** 10.3390/molecules29102177

**Published:** 2024-05-07

**Authors:** Zhuowei Tu, Alei Geng, Yuhua Xiang, Anaiza Zayas-Garriga, Hao Guo, Daochen Zhu, Rongrong Xie, Jianzhong Sun

**Affiliations:** 1 Biofuels Institute, School of the Environment and Safety Engineering, Jiangsu University, Zhenjiang 212013, China; zwtu99@hotmail.com (Z.T.); mr.linyue@outlook.com (Y.X.); anaiza.zayas10@gmail.com (A.Z.-G.); gh15036951363@hotmail.com (H.G.); dczhucn@ujs.edu.cn (D.Z.); rrxie@ujs.edu.cn (R.X.); 2 Changzhou Engineering and Technology Institute, Jiangsu University, Changzhou 214153, China

**Keywords:** lignin linkages, *Klebsiella aerogenes*, lignocellulose, catalytic mechanism

## Abstract

Lignin, the largest non-carbohydrate component of lignocellulosic biomass, is also a recalcitrant component of the plant cell wall. While the aerobic degradation mechanism of lignin has been well-documented, the anaerobic degradation mechanism is still largely elusive. In this work, a versatile facultative anaerobic lignin-degrading bacterium, *Klebsiella aerogenes* TL3, was isolated from a termite gut, and was found to metabolize a variety of carbon sources and produce a single kind or multiple kinds of acids. The percent degradation of alkali lignin reached 14.8% under anaerobic conditions, and could reach 17.4% in the presence of glucose within 72 h. Based on the results of infrared spectroscopy and 2D nuclear magnetic resonance analysis, it can be inferred that the anaerobic degradation of lignin may undergo the cleavage of the C-O bond (β-O-4), as well as the C-C bond (β-5 and β-β), and involve the oxidation of the side chain, demethylation, and the destruction of the aromatic ring skeleton. Although the anaerobic degradation of lignin by TL3 was slightly weaker than that under aerobic conditions, it could be further enhanced by adding glucose as an electron donor. These results may shed new light on the mechanisms of anaerobic lignin degradation.

## 1. Introduction

Lignocellulose represents the most abundant renewable source of carbon on the planet. Every year, more than 200 billion tons of non-food lignocellulosic biomass is produced [1], which is a valuable source of organic carbon that may substitute petroleum in the synthesis of various organic chemicals. Lignocellulosic biomass is generally composed of lignin (26–31%), hemicellulose (25–32%), and cellulose (41–46%). As the largest non-carbohydrate fraction of lignocellulose, the utilization of lignin has attracted increasing attention. Lignin is a complex racemic aromatic heteropolymer, which is made up of three differently methylated hydroxycinnamic alcohol monomers: *p*-coumaryl alcohol (H), coniferyl alcohol (G), and sinapyl alcohol (S) linked by various C-O and C-C bonds [2,3]. The high phenyl-ring structure endows lignin with unique physiochemical characteristics, and could be converted into a series of value-added chemicals [4,5].

However, lignin is highly resistant to chemical and biological degradation. It not only confers mechanical resistance to wood, but also leads to the non-specific adsorption of cellulolytic as well as hemicellulolytic enzymes during the saccharification of lignocellulosic biomass, and is associated with enzyme inhibition [6]. This recalcitrant polymer is usually found in the secondary cell wall of plant cells, where it combines with hemicellulose to create an amorphous matrix that embeds cellulose protofibrils and inhibits their biodegradation [7]. Lignin’s recalcitrance has hindered the conversion of biomass into bioproducts; nevertheless, exploring strategies for lignin degradation may allow new industrial technologies to improve the economics of lignocellulosic biorefineries [8].

In the natural world, a series of fungi and bacteria are able to break down lignin, with bacteria being the versatile microbes for this process, which is also an essential part of global carbon recycling. Compared to fungi, bacteria possess faster growth rates and more diverse and efficient metabolic pathways for the breakdown of lignin aromatic compounds [4,9,10]. Plenty of research focuses on the aerobic lignin transformation by micro-organisms, which primarily use free radicals and enzymes such as peroxidase as well as laccase to oxidatively tear lignin apart. Nevertheless, the oxygen supply is a major cost in the fermentation industry, which results in oxygen-free anaerobic fermentation becoming of great potential in industrial application. There are relatively few studies on the anaerobic degradation of lignin by individual bacterial strains, whose specific mechanism is still ambiguous. While some anaerobic microbes just use the methyl groups in lignin [11], others may need electron donors.

The anaerobic lignin degradation process is substantial and may involve an array of enzymes as well as pathways. A series of anaerobic lignin-degrading bacteria have been isolated. *Acetoanaerobium* sp. WJDL-Y2, which was isolated from pulp and paper mill sludge, demonstrated kraft lignin degradation and modification capabilities [12]. Rashid et al. validated the lignin-degrading and gas-producing abilities in four anaerobic bacterial strains isolated from municipal-solid-waste-containing soil [13]. The genomic profiles of some anaerobic lignin-degrading bacteria have also been revealed. *Enterobacter lignolyticus* SCF1, a facultative anaerobic bacterium, utilizes the 4-hydroxybenzoate degradation pathway, peroxidase/peroxygenase system, glutathione biosynthesis, and upregulation of glutathione S-transferase (GST) proteins for lignin degradation [14]. *Klebsiella* sp. BRL6-2 possesses genes associated with a complete protocatechuate pathway, six putative peroxidase genes, two lactate dehydrogenase genes, and two catalase genes [15]. *Tolumonas lignolytica* BRL6-1T harbors various lignin degradation pathways; hence, it could enzymatically cleave the β-aryl ether bonds, depolymerizes lignin through extracellular peroxidases, and breaks down lignin-derived aromatic compounds [16]. Chaput et al. isolated a novel anaerobic lignin-degrading species, *Sodalis ligni*, from forest soil, and also found a series of lignin and aromatic compound degradation/metabolism enzymes in its genome [17]. Despite the knowledge of the genomic information of these bacteria, the anaerobic degrading mechanism of lignin is still not fully understood.

In the present study, a facultative anaerobic lignin-degrading bacterium, *Klebsiella aerogenes* TL3, was isolated from a termite gut, the latter of which is considered to be the most efficient bioreactor of lignocellulose, as well as a reservoir of lignocellulolytic microbes. TL3 is capable of anaerobically degrading alkali lignin as well as several other carbon sources to produce a single kind or multiple kinds of acids under anaerobic or aerobic conditions. Specific studies on the changes of the physicochemical properties of lignin and the degradation capacity of TL3 revealed a unique degradation pattern, which might shed new light on the mechanisms of the anaerobic decomposition of lignin.

## 2. Results

### 2.1. Growth of TL3 on Various Carbon Sources

A variety of substrates can be effectively utilized by TL3 to produce multiple compounds (Table 1). Surprisingly, the aerobic metabolism of glucose led to an exclusive product of propanoic acid (3.11 g/L), while the aerobic metabolism of xylose gave a single product of butyric acid (1.81 g/L). In addition, no accumulation of acids was found in the degradation of avicel, lignin, and corn cob by TL3 under aerobic condition, but the bacterial growth was clearly observed. By comparison, the degradation of different carbon sources by TL3 under anaerobic conditions produced various types of acids. The richest variety of acids was generated by glucose degradation, of which propionic acid was the most abundant at 2.7 g/L. Of particular note, TL3 was able to degrade corn cob and alkali lignin under anaerobic conditions to produce lactic acid and succinic acid.

### 2.2. The Lignin Degradation Curve

**Figure 1:**
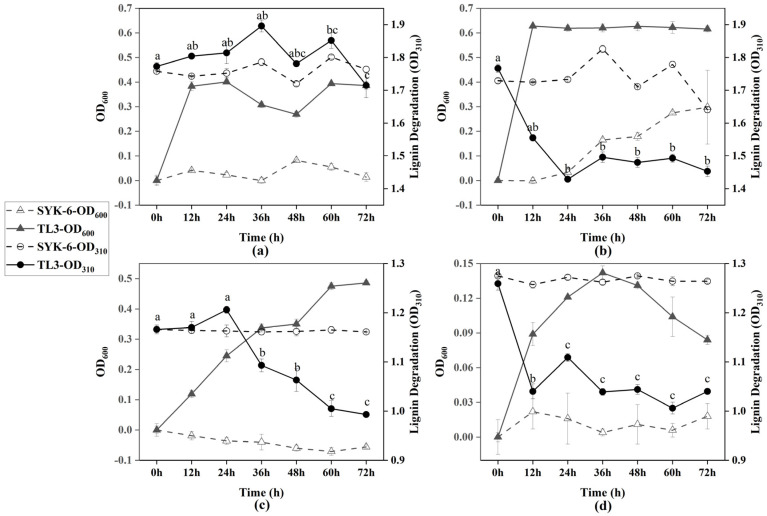
Growth curve of TL3 and anaerobic lignin degradation under different conditions: (**a**) mMRS medium supplemented with 1 g/L lignin; (**b**) mMRS medium supplemented with 1 g/L lignin and glucose; (**c**) MSM medium supplemented with 1 g/L lignin; and (**d**) MSM medium supplemented with 1 g/L lignin and glucose. Error bars showing the standard error of triplicate samples. Different letters indicate statistically significant differences among groups at *p* < 0.05.

To determine the growth characteristics and the capability of lignin degradation by strain TL3 under different conditions, cultures were grown for 3 days in mMRS or MSM media with lignin as the carbon source, with or without the addition of glucose. A typical lignin-degrading strain, *Sphingobium* sp. SYK-6, was used as a control strain. Optical densities (OD_600_ and OD_310_) were measured every 12 h. Lignin contents correlated with the absorbance at 310 nm, where a decrease in absorbance indicates a reduction in the concentration of soluble phenolic and polymeric lignin [16]. As shown in Figure 1a, without the addition of glucose, TL3 was able to grow in the mMRS medium (OD_600_ reached 0.401), but there was almost no change in OD_310_, indicating that TL3 could grow in this complex medium with minimal lignin consumption. By comparison, TL3 was indeed able to grow in the mMRS medium in the absence of lignin or glucose, reaching a maximum OD_600_ of 0.198 (Supplementary Materials: Figure S1). From Figure 1b, it can be observed that TL3 grew rapidly in the mMRS medium supplemented with glucose, reaching a maximum OD_600_ of approximately 0.616 within 12 h, with the percent degradation of lignin reaching approximately 17.8%. Interestingly, as shown in Figure 1c, TL3 was able to grow in the MSM medium with lignin as the sole carbon source, reaching a maximum OD_600_ of 0.486 within 72 h, and achieving a percent degradation of 14.8%. At this point, the color of the medium changed from brown to black-green. In Figure 1d, TL3 reached its highest OD_600_ (0.142) in the MSM medium supplemented with glucose at 36 h, and the percent degradation of lignin was approximately 17.4%. In most cases, the control strain, SYK-6, did not grow well under anaerobic conditions, except for that in the rich medium supplemented with glucose (Figure 1b).

### 2.3. FTIR Plots of Lignin Degradation by TL3 under Various Conditions

**Figure 2:**
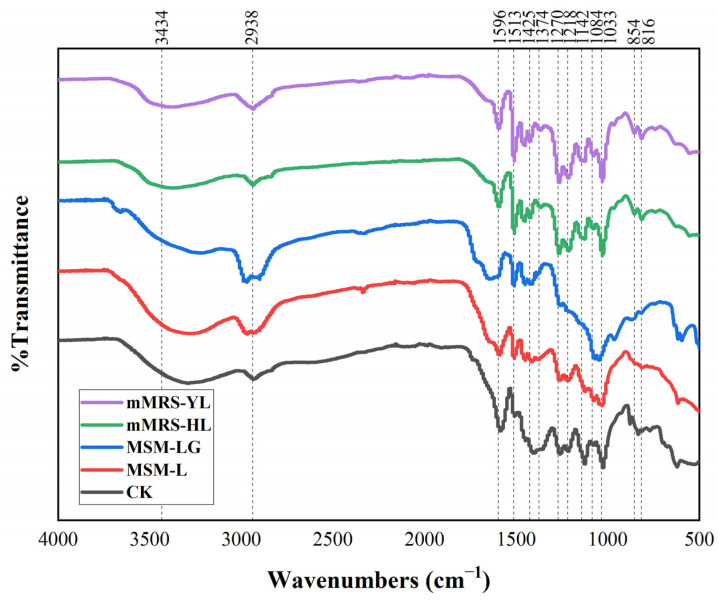
The FTIR spectra of lignin before and after treatment by TL3. CK, untreated alkali lignin; MSM-L, lignin was anaerobically treated with TL3 in MSM media; MSM-LG, lignin was anaerobically treated with TL3 in MSM media supplemented with 1 g/L glucose; mMRS-HL, lignin was aerobically treated with TL3 in mMRS media; and mMRS-YL, lignin was anaerobically treated with TL3 in mMRS media.

In order to understand the structural changes in lignin by bacterial treatment, FTIR spectroscopy was employed to analyze lignin samples before and after degradation. Figure 2 depicts the FTIR spectra of alkali lignin before and after treatment with strain TL3 under different conditions. Clear differences in the characteristic peaks of lignin were observed before and after degradation, primarily ranging between 1800 cm^−1^ and 800 cm^−1^, with the most significant variations observed when a little glucose was added into the lignin-mineral medium as an electron donor. Across all conditions, the absorption peaks at 1596 cm^−1^, 1425 cm^−1^, 1374 cm^−1^, and 1142 cm^−1^ exhibited a decrease, suggesting potential side-chain oxidation, methyl removal, and aromatic-ring-framework modification (Table S1). However, the mMRS-HL and mMRS-YL spectra were quite similar to CK, suggesting very little modification in the lignin structure has occurred under these conditions, and the growth of TL3 might primarily rely on the complex nutrition factors in the medium, which is consistent with the results of Figure 1a and Figure S1. By comparison, in the MSM medium, significant changes occurred in the lignin absorption peaks between 1270 cm^−1^ and 816 cm^−1^ under anaerobic conditions, particularly when glucose was added, where the peaks at 1218 cm^−1^, 1142 cm^−1^, and 1084 cm^−1^ almost disappeared, indicating the extensive disruption of the β-O-4 linkage. The increased absorption at 1033 cm^−1^ indicated a higher C_α_-ether content after TL3 treatment. The peaks at 854 cm^−1^ and 816 cm^−1^ nearly disappeared, while those at 3434 cm^−1^ and 2938 cm^−1^ increased, suggesting the modification of lignin side-chains by strain TL3. Overall, lignin degradation was more effective in the MSM medium compared to the mMRS medium, especially when glucose was supplemented as an electron donor in the MSM medium. Hence, studies were primarily carried out using the MSM medium hereafter.

### 2.4. GC-MS Analysis of the Metabolites

In order to understand the metabolic profiles of lignin by TL3, we conducted a GC-MS analysis to identify the products of lignin decomposition in the MSM medium. Untreated lignin served as a control group. The chromatographic peaks were identified and assigned according to the National Institute of Standards and Technology (NIST) library. The peak areas from the chromatograms were used to determine the amount of each aromatic degradation product. As shown in Table 2, the control sample had quite a few extractive chemicals, while a series of new chemicals showed up in the TL3-treated sample, suggesting a substantial decomposition of lignin. These newly appeared compounds included aliphatic hydrocarbon, aromatic hydrocarbons, aliphatic acid, phenolic acid, phenol, ester, etc. Furthermore, with the addition of glucose as an electron donor (TL3 + Glucose treatment), lignin degradation became more thorough, and an array of end metabolites with a small molecular weight accumulated, such as propanoic acid and formic acid. It is noteworthy that there were almost no intermediates such as ferulic acid and *p*-coumaric acid in the degradation products, but, rather, mainly several kinds of end products.

### 2.5. 2D-HSQC NMR Analysis of the Residual Lignin

**Figure 3:**
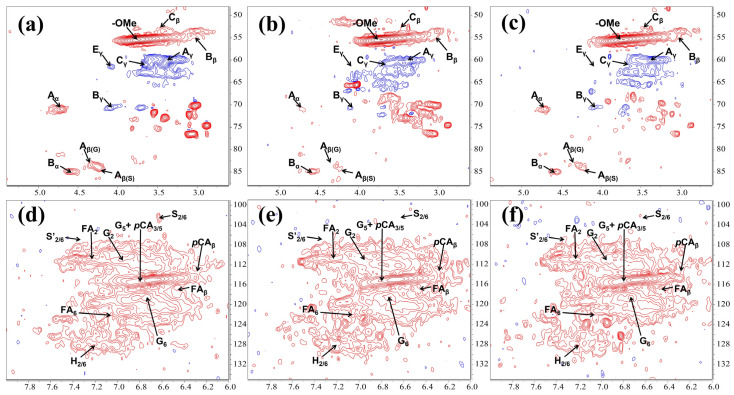
2D ^1^H-^13^C HSQC NMR spectra of lignin before and after treatment by TL3: (**a**–**c**) oxygenated aliphatic region; and (**d**–**f**) aromatic region. (**a**,**d**) Untreated lignin; (**b**,**e**) lignin was treated by TL3 in MSM media under aerobic condition; and (**c**,**f**) lignin was treated by TL3 in MSM media supplemented with 1 g/L glucose under anaerobic condition.

The cleavage of the lignin C-O-C bond is crucial in the bacterial lignin degradation process. In this study, lignin linkages such as β-O-4, β-β, and β-5 showed varying degrees of cleavage after treatment with TL3 (Figure 3 and Figure S2 and Table S2). The contours in the NMR spectra are used to determine changes in lignin linkages; a weakening or strengthening of the contours indicates a change in bond energy. As depicted in Figure 3a–c, the β-O-4 aryl ether linkage (A_α_, A_β(G)_, and A_β(S)_) was almost completely degraded under aerobic conditions, and was more pronounced than that under the optimized anaerobic degradation conditions (with the addition of glucose as an electron donor). The partial cleavage of the β-β (B_α_, B_β_, and B_γ_) and β-5 (C_β_, and C_γ_) linkages was also observed, with a similar effectiveness both under the aerobic and the anaerobic conditions. In Figure 3d–f, it can be observed that the syringyl (S_2/6_) units were completely degraded both under aerobic and anaerobic conditions, with oxidation occurring under aerobic conditions; while the *p*-coumaric acid (*p*CA_3/5_, *p*CA_β_) and the ferulic acid (FA_2_, FA_6_, FA_β_) structure showed almost no change before and after treatment. Altogether, these results suggested that the lignin degradation level under aerobic conditions was slightly higher than that under the optimized anaerobic conditions (with an electron donor).

## 3. Discussion

This study presents the profiles of lignin degradation both under aerobic and anaerobic conditions by a newly isolated bacterium, *Klebusiella aerogenes* TL3, which was isolated from the gut of a termite, *Odontotermes formosanus*. The degradation process led to the breakdown of key lignin linkages such as β-O-4, β-β, and β-5, resulting in the production of low-molecular-weight aromatic compounds and organic acids. TL3 was also capable of degrading some monosugars as well as some polysaccharides, including cellulose and natural corn cob. These findings suggested that TL3 is a versatile bacterium, which may shed light on the knowledge of lignocellulose decomposition by facultative anaerobic bacteria.

The versatile degradation capability of TL3 is unique among known bacteria. Previously, it has been reported that *Klebsiella* sp. strain BRL6-2 was capable of utilizing various substrates under aerobic and anaerobic conditions without discrimination, including D-cellobiose, D-mannose, Tween 20, D-glucose, and D-fructose [15]. Many anaerobic cellulolytic bacteria, such as *Thermoanaerobacter* spp., *Clostridium* spp., and *Caldicellulosiruptor* spp., are good at decomposing cellulose, but some of them do not utilize xylan as well as xylose, and few of them can assimilate lignin [18]. In contrast, TL3 can utilize cellulose, lignin, and some monosugars, suggesting its powerful metabolic capability (Table 1). Known potential lignin-degrading bacteria belong to the phyla *Proteobacteria*, *Actinobacteria*, *Firmicutes*, and *Bacteroidetes* [19], and most of them decompose lignin through extracellular oxidases, which require the availability of oxygen [20], while many anaerobic lignin-degrading bacteria do not metabolize cellulose or xylan [14,21]. In comparison to the aforementioned studies, TL3 exhibits growth on lignin both under aerobic and anaerobic conditions, making it a versatile and unique strain for the degradation of lignocellulose.

The anaerobic lignin degradation process is featured by a quick yet incomplete decomposition, and could probably be enhanced by the addition of specific electron acceptors. In the presence of 0.2% glucose, the concentration of lignin decreased from 0.14% to approximately 0.092% after anaerobic treatment by *Tolumonas lignolytica* BRL6-1 [16]. *Acetoanaerobium* sp. WJDL-Y2 can degrade approximately 24.9% of kraft lignin under anaerobic conditions [12]. *Enterobacter lignolyticus* SCF1 can anaerobically degrade 56% of lignin when supplemented with xylose (with 0.8% xylose and 0.05% lignin in the culture medium), but it cannot grow on liquid media with lignin as the sole carbon source [14]. The degradation of lignin in the aforementioned results requires the presence of electron acceptors (such as glucose, xylose, etc.) to achieve efficient lignin degradation. Similarly, in this study, the addition of glucose to the culture medium promoted lignin degradation from 14.8% to 17.4% (Figure 1c,d). In the aerobic degradation of lignin with different concentrations by *Pseudomonas* sp. Hu109A, the highest percent degradation of 88.13% was achieved at a lignin concentration of 0.5 g/L, while the percent degradation was approximately 60% at a lignin concentration of 1 g/L [22]. It is known that the degradation rate decreases with increasing lignin concentration, partly due to the biotoxicity of lignin to bacteria and the inhibitory effect of the degradation products on bacterial growth. SCF1 and WJDL-Y2 have a high anaerobic degradation efficiency and can reach maximum conversion within approximately 48 h [12,14], while the aerobes degraded lignin continuously at a slow pace for 7–28 days [22]. The higher conversion of lignin under aerobic conditions may correlate with its longer time span of degradation than that under anaerobic conditions.

The cleavage of various linkages is crucial for the process of lignin decomposition. The predominant linkages in lignin are β-O-4 aryl ether linkages, which account for 45–60% of the total linkages, while other linkages in natural lignin include β-5 phenylcoumaran (6–12%), 5-5′ biphenyl (9–22%), β-β′ resinol (2–4%), β-1 dibenzodioxocin (1–9%), and 4-O-5′ biphenyl ether (1–7%) linkages [23]. The aryl ring cleavage, carboxylation, and demethylation happened during the anaerobic degradation of kraft lignin by strain WJDL-Y1 [24]. Thomas et al. treated various natural lignocellulosic fibers with several anaerobic fungi, revealing the loss of both β-O-4 and β-5 linkages, the significant hemicellulose reshaping, and the removal of dangling units such as ferulic acid, *p*-coumaric acid, and *p*-hydroxybenzoic acid [25]. Apparently, TL3 treatment led to more kinds of linkage reduction (β-O-4, β-5, and β-β linkages), with the syringyl units being completely removed, and the presence of an electron acceptor promotes the linkage cleavage (Figure 3). In addition, the extent in the cleavage of lignin linkages by TL3 was significantly higher under aerobic conditions than that under anaerobic conditions (Figure 3b,c). This was also evidenced by the fact that more types of lignin linkages were disrupted by some aerobic lignin degraders. For example, an NMR analysis revealed that the β-O-4, β-β, β-5, and β-1 linkages were broken during the aerobic degradation of kraft lignin by the secretome of *P. putida* KT2440 [26]. In addition, an introduction of H_2_O_2_ into the secretome activated peroxidases, resulting in the increased cleavage of β-O-4 and C-C (β-β and β-1) linkages, with a further enhancement on the cleavage of these linkages upon the addition of Mn^2+^, suggesting the power of hydroxy radicals in linkage cleavage, which might be absent in the anaerobic degradation process. However, the removal of aromatic rings by TL3 is less pronounced (Figure 3d–f), possibly due to the lack of relevant degrading enzymes.

The metabolic intermediates and end products were also different from other known lignin degraders. Compared to fungi, bacteria exhibit lower lignin-degrading activity, but their versatile metabolic capabilities enable them to degrade various aromatic compounds derived from lignin [27]. Previous studies have identified compounds such as benzoic acid, vanillin, acetosyringone, 2-methylbenzoic acid, coumaric acid, and vanillic aldehyde in the extractives of kraft lignin [24,28]. Vanillin and coumaric acid are present in almost all lignin degradation products by aerobic microbes, with the main products of aerobic lignin degradation by Hu109A also including dibutyl phthalate, acetosyringone, 2,4-di-tert-butylphenol, and eugenol [22]. This differs significantly from our results, where lignin monomer substances such as syringic acid and guaiacol, as well as other O-demethylated derivatives such as gallic acid and protocatechuic acid, could not be detected in the treated samples (Table 2), perhaps because of their different metabolic pathways. The lignin degradation products by TL3 include derivatives of various lignin monomeric structures such as *p*-xylene, phthalic acid, and phenylpropionic acid, which is similar to the results of a previous study on lignin degradation by the anaerobic lignin-degrading bacterium, *Trabulsiella* sp. [29], but different from another one, *Acetoanaerobium* sp. WJDL-Y2 [12]. This indicates the extreme diversity in microbial selectivity and the preference for lignin bond cleavage and aromatic compound metabolism, resulting in vastly different degradation products among different bacteria [30].

In conclusion, it is evident that *K. aerogenes* TL3 is a versatile facultative anaerobic lignin-degrading bacterium capable of simultaneously degrading the main components of lignocellulose. Its efficiency in anaerobic lignin degradation was a little weaker than that under aerobic conditions, with a percent degradation of up to 17.4% in the presence of glucose. TL3 primarily disrupts lignin side chains and cleavages the β-O-4, β-5 and β-β′ linkages, which, accordingly, leads to the production of small-molecule organic acids such as propionic acid, lactic acid, and succinic acid. The difference in lignin degradation by TL3 between aerobic and anaerobic conditions may help better understand the process and mechanisms of lignocellulose decomposition in nature. Moreover, this study may shed new light on the knowledge of the mechanism of anaerobic lignin degradation, and pave a way toward the efficient anaerobic conversion of biomass.

## 4. Materials and Methods

### 4.1. Strain and Media

TL3 was isolated using a modified deMan Rogosa Sharpe (mMRS) medium [31] supplemented with 1 g/L lignin instead of glucose from the gut of a termite (*Odontotermes formosanus*), which was collected from the campus of Jiangsu University by us. TL3 was deposited at China General Microbiological Culture Collection Center (CGMCC 1.61912), and identified as *Klebusiella aerogenes* because it shared 99.8% 16S rDNA similarity with the known members of this species (GenBank accession: PP577623). Luria-Bertani (LB) medium contains (g/L) tryptone 10 g, Yeast extract 5 g, and NaCl 5 g. The mMRS medium contains (g/L) tryptone 1 g, YE 2 g, beef extract 2 g, glucose 5 g, KH_2_PO_4_ 1.5 g, K_2_HPO_4_ • 3H_2_O 2.9 g, sodium acetate 1 g, triammonium citrate 0.5 g, MgCl_2_ • 6H_2_O 0.2 g, CaCl_2_ • 2H_2_O 75 mg, and MnSO_4_ 0.05 g. After boiling and nitrogen-flushing for 10 min, L-cysteine was added into the mMRS medium as a reducing reagent at a final concentration of 0.5 g/L. The mineral salt medium (MSM) contains 100 mM KH_2_PO_4_, 75 mM KOH, 15 mM (NH_4_)_2_SO_4_, 1 mM MgSO_4_, 3.9 µM FeSO_4_, and 1 g/L alkaline lignin (CAS8068-05-1, Catalog number 370959, Sigma–Aldrich, St. Louis, MO, USA). One gram per liter of glucose was added into the MSM medium as an electron donor where necessary.

### 4.2. Metabolic Characterization of Different Carbon Sources

In order to determine the fermentation profiles of strain TL3, fresh TL3 cultures were firstly transferred to 15 mL of mMRS medium at 1% inoculum and incubated anaerobically at 37 °C and 180 rpm for 24 h. Then, the TL3 culture was inoculated into mMRS medium supplemented with 10 g/L various carbon sources such as glucose, avicel, xylose, corn cob (60 mesh), lignin, etc., and grew anaerobically or aerobically at 37 °C and 180 rpm for 24 h. The metabolites were determined using high-performance liquid chromatography (HPLC) as described below: The fermentation broth was centrifuged at 13,000 rpm, and the supernatant was filtered and acidified with 0.2 M sulphuric acid. The HPLC was equipped with an Aminex HPX-87H column and a refractive index detector, utilizing 4 mM sulphuric acid as the mobile phase at a flow rate of 0.4 mL/min, and the temperature of the column was maintained at 50 °C.

### 4.3. Time Courses of Bacterial Growth and Lignin Degradation under Anaerobic Conditions

For the determination of bacteria growth and lignin degradation under anaerobic conditions, TL3 and the control strain were grown in the MSM medium or the mMRS medium, with or without the supplement of 1 g/L glucose. One milliliter of fermentation broth was taken every 12 h, and the optical density was measured at 600 nm, deionized water was used as blank control, and the above experiments were conducted in three parallel experiments. Lignin concentration was measured by retrieving 1 mL of culture from anaerobic septum bottles, diluting 1:2 in distilled water, filtering out cells, and measuring the absorbance at 310 nm as described by Billings et al., 2015 [16]. For statistical analysis, Turkey’s test was carried out using Origin™ Pro 9.0 software.

### 4.4. Fourier-Transform Infrared Spectroscopy (FTIR) Analysis

After three days of aerobic or anaerobic incubation, the fermentation broth was centrifuged at 13,000 rpm to remove the cells. Subsequently, the supernatant was acidified with 0.5 M sulfuric acid to precipitate the alkali lignin, which is water-insoluble under acid condition and water-soluble at weak acid condition or at even higher pHs. Samples were washed repeatedly with acidified pure water and dried in an oven at 60 °C. The lignin FTIR spectra in attenuated total reflection (ATR) mode were collected by the potassium bromide tablet method by taking a certain amount of dried, treated, or untreated lignin samples, respectively. The lignin samples were mixed with potassium bromide at a mass ratio of 1:100, ground to 200 mesh powder, and prepared into thin slices of uniform thickness. The FTIR spectra were collected, with pure potassium bromide as the background. The wavenumber range was 4000–400 cm^−1^, and the number of scans was 64 with a resolution of 1 cm^−1^.

### 4.5. Gas Chromatography–Mass Spectrometry (GC-MS) Analysis

Lignin was anaerobically degraded by TL3 in 50 mL of MSM medium with or without the supplement of glucose for 3 days; while the control sample was not inoculated with bacteria. Samples were centrifuged at 13,000 rpm for 15 min to remove biomass. Then, the supernatants were acidified to pH 2–3 with 6 M HCl and thoroughly extracted afterward with 150 mL of ethyl acetate. The organic layer of the extraction mixture was collected and reduced to 10 mL by rotary evaporation at 37 °C and dewatered by anhydrous Na_2_SO_4_ to remove moisture. Then, 100 µL of the organic layer was silylated after the evaporation of the solvent under nitrogen stream. For the silylation procedure, 100 μL dioxane and 10 μL pyridine were added into samples and vortexed in glass tubes followed by silylation, which was performed with 50 μL of N,O-bis(trimethylsilyl)trifluoroacetamide (BSTFA; Sigma-Aldrich, St. Louis, MO, USA). The mixture was heated in a water bath at 80 °C for 45 min with periodic shaking to dissolve the residues. An aliquot of 1 mL of silylated mixture was injected into a gas chromatography–mass spectrometry (GC-MS) system (AntoSystem XL GC-TurboMass; PerkinElmer, Waltham, MA, USA). The analytical column was connected to the system was a PE-5MS capillary column (20 m × 0.18 mm internal diameter, 0.18 mm film thickness). A solvent delay of 3.0 min was selected. In the full-scan mode, electron ionization mass spectra in the range of 30–550 (*m*/*z*) were recorded at electron energy of 70 eV. All standard monomeric phenolics compounds (1 mg) were derivatized and chromatographed as above. In order to identify the low-molecular-weight lignin-related compounds as trimethylsilyl (TMS) derivatives derived from bacterial treatment, their mass spectra were compared with that of the data of GC-MS spectral library (Wiley, NIST) available in the instrument.

### 4.6. Nuclear Magnetic Resonance (NMR) Analysis

The 2D-^1^H-^13^C heteronuclear single-quantum coherence (HSQC) NMR spectra were obtained using a Bruker Avance III HD 500 MHz spectrometer operating at a frequency of 125.12 MHz for the 13C nucleus as described by Ji et al. (2021) [32]. Thirty to fifty milligrams of the dry lignin samples were dissolved in 0.6 mL deuterated dimethylsulfoxide (DMSO)-d6 and the spectra were collected at 298 K. A standard Bruker adiabatic HSQC pulse sequence (hsqcetgpsisp2.2) was used with the following spectra acquisition condition: 1.0 s pulse delay, 64 scans, 1024 data points for ^1^H, 256 increments for ^13^C, and a 1JC–H of 145 Hz. The ^1^H and ^13^C spectral widths are 13.0 and 220.0 ppm, respectively. The central DMSO solvent peak (δ^13^C/δ^1^H = 39.5/2.49 ppm) was used for chemical shifts calibration. HSQC spectra were processed and analyzed with Mestrenova (version 12.0.2) with a matched cosinebell apodization and 2 × zero filling in both dimensions.

## Figures and Tables

**Table 1:** Fermentation products by TL3 on different carbon sources.

Methods	Fermentation Products (g/L)	Carbon Sources				
		Avicel	Glucose	Xylose	Corn Cob	Lignin
Anaerobic	Lactic Acid	ND ^a^	0.73 ± 0.01	0.85 ± 0.02	0.23 ± 0.01	0.13 ± 0.02
	Acetic Acid	ND	1.25 ± 0.02	0.52 ± 0.01	ND	ND
	Succinic Acid	0.64 ± 0.03	1.07 ± 0.00	1.69 ± 0.01	0.82 ± 0.02	1.43 ± 0.00
	Propanoic acid	0.26 ± 0.02	2.70 ± 0.03	0.32 ± 0.03	ND	0.26 ± 0.01
	Butyric Acid	ND	1.38 ± 0.02	ND	ND	ND
Aerobic	Lactic Acid	ND	ND	ND	ND	ND
	Acetic Acid	ND	ND	ND	ND	ND
	Succinic Acid	ND	ND	ND	ND	ND
	Propanoic acid	ND	3.11 ± 0.01	ND	ND	ND
	Butyric Acid	ND	ND	1.81 ± 0.02	ND	ND

^a^ “ND”, not detected.

**Table 2:** Products of lignin degradation in different treatments.

NO.	RT (min)	Compounds	Treatment		
			Control	TL3	TL3 + Glucose
1	1.344	Acetic acid, (propylthio)-	−	+	−
2	2.879	3-Ethylheptanoic acid	−	−	+
3	3.256	Propanoic acid, ethyl ester	−	+	+
4	3.400	Formic acid, butyl ester	−	+	+
5	6.400	*p*-Xylene	−	+	−
6	9.766	L-(+)-Lactic acid	−	+	−
7	11.921	1,3-Butanediol, diacetate	−	+	−
8	13.398	Benzaldehyde, 3,4-dimethyl-	+	+	−
9	13.944	Oxalic acid, 6-ethyloct-3-yl isobutyl ester	−	+	−
10	14.132	Heptadecane, 8-methyl-	+	+	−
11	15.287	Butyric acid, 4-pentadecyl ester	−	+	−
12	15.587	Butanoic acid, octyl ester	−	+	−
13	17.032	Octacosane	+	+	−
14	17.288	Phenol, 2,5-bis(1,1-dimethylethyl)-	−	+	+
15	18.943	Pentadecane, 2,6,10-trimethyl-	−	+	−
16	19.532	Hexane, 2,4-dimethyl-	−	+	−
17	19.543	Heneicosane	+	+	−
18	20.786	3-Vanilpropanol, bis(trimethylsilyl)-	−	+	−
19	21.286	Phthalic acid, isobutyl undecyl ester	−	+	+
20	21.798	Heptacosane	−	+	−
21	21.863	7,9-Di-tert-butyl-1-oxaspiro(4,5)deca-6,9-diene-2,8-dione	+	−	−
22	21.942	Methoxyacetic acid, 3-tridecyl ester	+	+	−
23	22.020	Benzenepropanoic acid, 3,5-bis(1,1-dimethylethyl)-4-hydroxy-, methyl ester	−	+	−
24	22.287	n-Hexadecanoic acid	+	+	−
25	22.565	Benzoic acid, 3-[(2,2-dimethyl-1-oxopropyl)amino]-	−	+	−
26	24.187	Hexadecanoic acid, 2-hydroxy-, methyl ester	−	+	−
27	24.508	Octadecanoic acid	+	+	−
28	26.109	Hexanedioic acid, bis(2-ethylhexyl) ester	−	+	−
29	26.309	Phenol, 2,2′-methylenebis[6-(1,1-dimethylethyl)-4-methyl-	−	+	+
30	26.686	2-Nonenoic acid, methyl ester	−	+	−
31	26.799	Benzenepropanoic acid, 3,5-bis(1,1-dimethylethyl)-4-hydroxy-, octadecyl ester	−	−	+
32	27.142	Hexadecanoic acid, 2-hydroxy-1-(hydroxymethyl)ethyl ester	−	+	−
33	27.253	1,2-Benzenedicarboxylic acid, dioctyl ester	−	+	−
34	27.797	Benzoic acid, 2,5-bis(trimethylsiloxy)-, trimethylsilyl ester	+	+	−
35	28.508	Butanedioic acid, 2-hydroxy-2-methyl-, dimethyl ester	−	+	−
36	28.819	Octadecanoic acid, 2-hydroxy-1-(hydroxymethyl)ethyl ester	−	+	−
37	28.897	Terephthalic acid, di(2-ethylhexyl) ester	−	+	−
38	28.842	Fumaric acid, dodecyl nonyl ester	−	+	−

Note: RT, retention time; “+” present; “−” absent.

## Data Availability

All data sets used and analyzed are available upon reasonable request.

## Supplementary Materials

The following supporting information can be downloaded at: https://www.mdpi.com/article/10.3390/molecules29102177/s1, Table S1: Assignment of FTIR spectra of lignin samples; Table S2: Main lignin 2D ^1^H-^13^C cross-peak assignments in the HSQC spectra. Figure S1: Growth of TL3 and SYK-6 in mMRS medium without additional carbon source. Figure S2: Main detected lignin linkages.
