# Supplementary material for: Lignin Degradation by Klebsiella aerogenes TL3 under Anaerobic Conditions
Source: Molecules. 2024 May 7;29(10):2177. doi: 10.3390/molecules29102177 (PMC11124209; doi:10.3390/molecules29102177)
Supplement: Supplementary file 1 [file molecules-29-02177-s001.zip › molecules-2976010-supplementary.pdf]

Supplementary Materials

# **Lignin degradation by *Klebsiella aerogenes* sp. TL3 under anaerobic conditions**

Zhuowei Tu [1], Alei Geng[1,2]*, Yuhua Xiang[1], Chunjing Zhang[1], Anaiza Zayas-Garriga[1], Hao Guo[1], Daochen Zhu[1], Rongrong Xie[1], and Jianzhong Sun[1]*

1 Biofuels institute, School of the environment and safety engineering, Jiangsu University, Zhenjiang, Jiangsu 212013, PR China

2 Changzhou Engineering and Technology Institute of Jiangsu University, Changzhou Jiangsu 214153, PR China

* Correspondence: galxj@ujs.edu.cn (A.G.);
jzsun1002@hotmail.com (J.S.)

**Table S1.** Assignment of FTIR spectra from control and degraded lignin samples.

| Functional group assignment | Wavenumbers ($cm^{-1}$) |
|---|---|
| O–H stretch in phenolic and aromatic | 3434 |
| C–H stretch in aromatic –OMe and side chain-$CH_2$ and $CH_3$ | 2938 |
| Aromatic ring vibrations and C=O stretching | 1596 |
| C=C stretching and a change in bond angle of the H–C–C of aromatic ring | 1513 |
| C–H deformation plus aromatic skeletal vibrations | 1425 |
| Symmetric deformation of C-H in methyl groups | 1374 |
| Vibrations of guaiacyl rings and stretching vibration of C–O | 1270 |
| C–C, C–O, and C=O stretching | 1218 |
| Vibration of aryl-ether bonds (β-O-4 linkage) | 1142 |
| C–O deformation at $C_\beta$ and aliphatic ether | 1084 |
| C–O deformation at $C_\alpha$ and aliphatic ether | 1033 |
| C–H out-of-plane deformation in positions 2,5 and 6 of G units | 854 |
| C–H out-of-plane deformation in positions 2,5 and 6 of G units | 816 |

**Table S2.** Main lignin 2D $^{1}H$-$^{13}C$ cross-peak assignments in the HSQC spectra.

| Label | δH (ppm) | δC (ppm) | Assignments |
|---|---|---|---|
| -OMe | 3.70 | 55.6 | C-H in methoxyls |
| $A_{\alpha}$ | 4.83 | 71.6 | $C_{\alpha}$-$H_{\alpha}$ in β-O-4' substructures (A) |
| $A_{\beta(G)}$ | 4.34 | 83.6 | $C_{\beta}$-$H_{\beta}$ in β-O-4' substructures linked to G units (A) |
| $A_{\beta(S)}$ | 4.10 | 85.8 | $C_{\beta}$-$H_{\beta}$ in β-O-4' substructures linked to S units (A) |
| $A_{\gamma}$ | 3.20-3.50 | 59.5-60.1 | $C_{\gamma}$-$H_{\gamma}$ in β-O-4' substructures (A) |
| $B_{\alpha}$ | 4.60 | 84.8 | $C_{\alpha}$-$H_{\alpha}$ in resinol (β-β') substructures (B) |
| $B_{\beta}$ | 3.05 | 55.1 | $C_{\beta}$-$H_{\beta}$ in resinol (β-β') substructures (B) |
| $B_{\gamma}$ | 3.87;4.10 | 70.8 | $C_{\gamma}$-$H_{\gamma}$ in resinol (β-β') substructures (B) |
| $C_{\beta}$ | 3.49 | 52.3 | $C_{\beta}$-$H_{\beta}$ in phenylcoumaran (β-5') substructures (C) |
| $C_{\gamma}$ | 3.65 | 61.9 | $C_{\gamma}$-$H_{\gamma}$ in phenylcoumaran (β-5') substructures (C) |
| $E_{\gamma}$ | 4.07 | 61.6 | $C_{\gamma}$-$H_{\gamma}$ in hydroxycinnamyl substructures (E) |
| $G_2$ | 6.95 | 111.0 | $C_2$-$H_2$ in guaiacyl units (G) |
| $G_5$ | 6.77 | 115.4 | $C_5$-$H_5$ in guaiacyl units (G) |
| $G_6$ | 6.74 | 118.8 | $C_6$-$H_6$ in guaiacyl units (G) |
| $S_{2/6}$ | 6.68 | 103.7 | $C_{2,6}$-$H_{2,6}$ in etherified syringyl units (S) |
| $S'_{2/6}$ | 7.30 | 106.3 | $C_{2,6}$-$H_{2,6}$ in oxidized syringyl units (S') |
| $H_{2/6}$ | 7.21 | 128.2 | $C_{2,6}$-$H_{2,6}$ in p-hydroxyphenyl substructures (H) |
| $FA_2$ | 7.26 | 111.3 | $C_2$-$H_2$ in ferulate units (FA) |
| $FA_6$ | 7.09 | 121.9 | $C_6$-$H_6$ in ferulate units (FA) |
| $FA_{\beta}$ | 6.39 | 116.5 | $C_{\beta}$-$H_{\beta}$ in ferulic acid (FA) |
| $pCA_{3/5}$ | 6.77 | 115.4 | $C_{3,5}$-$H_{3,5}$ in *p*-coumarate units (*p*CA) |
| $pCA_{\beta}$ | 6.23 | 113.7 | $C_{\beta}$-$H_{\beta}$ in *p*-coumarate (*p*CA) |

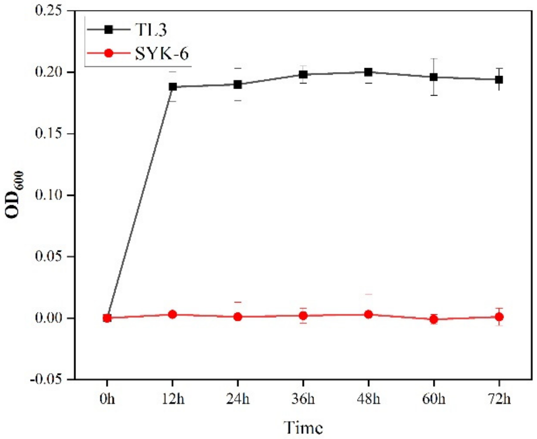


**Figure S1.** Growth of TL3 and SYK-6 on mMRS medium without additional carbon source.

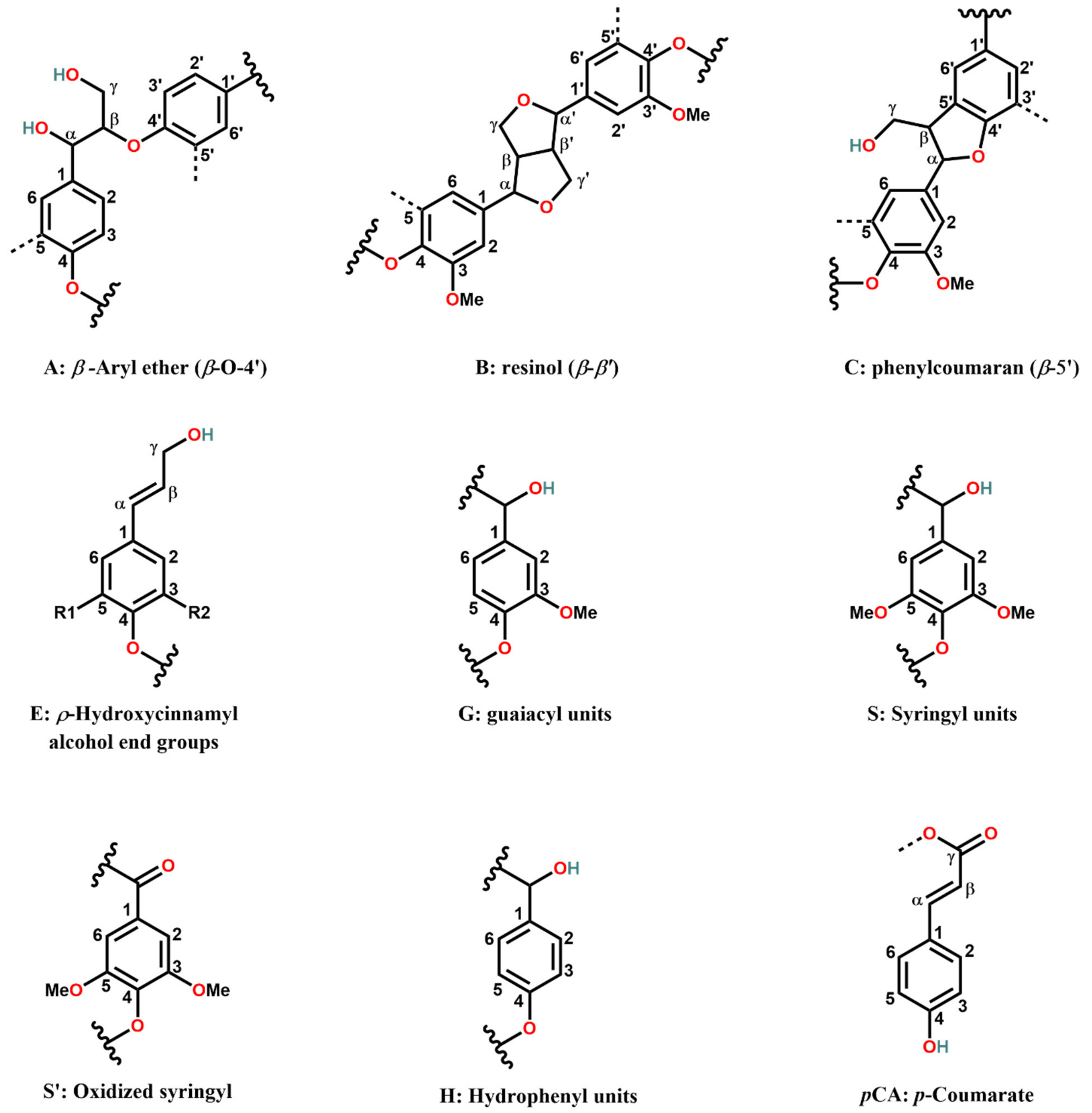


**Figure S2.** Main detected lignin linkages.
